# Incorporating Dermatologic Clinical Research Into Private Practice: A Review

**DOI:** 10.7759/cureus.57733

**Published:** 2024-04-06

**Authors:** Kristin N Slater, David Fivenson

**Affiliations:** 1 Dermatology, Lincoln Memorial University-DeBusk College of Osteopathic Medicine, Harrogate, USA; 2 Dermatology, Fivenson Dermatology, Ann Arbor, USA

**Keywords:** incorporating dermatologic clinical research, establishing research in practice, drug trials in private practice, dermatology, research in private practice, clinical drug trials, private practice, dermatologic clinical trials, clinical trials, clinical research

## Abstract

Clinical research is beneficial for the continued progression of medicine and the larger body of scientific knowledge. Clinical research can be incorporated into a range of settings, ranging from larger learning institutions to small private practices. With the need for continued advancement of the development of pharmaceutical interventions as well as other forms of clinical understanding, it is advantageous to create an environment where smaller, private practices feel comfortable and guided in establishing clinical research. It can be difficult to find the best methods to incorporate in-house clinical research. This review aims to address this gap in the literature, making the establishment of clinical trials, specifically clinical drug trials, more accessible in dermatology for private practices.

## Introduction and background

Clinical research has been noted to exist throughout history in differing forms and has continued to evolve [1]. The first modern clinical trial with a documented control group was conducted by Dr. James Lind [1]. Over time, clinical research has expanded into the extensive field that we see today, equipped with safeguards, rules, and guidelines to protect both the patient populations and the integrity of the datasets being studied [1]. Clinical research is a broad term describing medical research that can involve a large array of interventional and non-interventional research types [2]. Clinical research has facilitated major advancements in medicine by creating breakthroughs in disease understanding, management, monitoring, and treatment [1-2]. Clinical drug trials are a form of research that has had a positive impact on medicine as a whole [1-2]. In dermatology, clinical research sites have played a role in facilitating the infrastructure to study numerous highly specific drug therapies and have increased our capability to treat otherwise difficult-to-treat complex/rare medical dermatologic conditions [1-5]. With the ongoing need for continued clinical research, it is important that there is accessibility and guidance for smaller private practices to know how to approach incorporating clinical research into the clinic setting. This literature review aims to fill that gap and provide guidance to private dermatologic practices looking to incorporate clinical research into their facilities. 

## Review

A starting point for incorporating clinical research is defining what clinical research is in a dermatologic setting. Clinical research is a broad term describing human research and can encompass a vast array of research, ranging from studying risk factors and disease pathophysiology to clinical drug trials, which study the efficacy and safety profile of drug treatment [2]. From a larger framework, clinical research can be divided into two categories: observational studies and experimental studies [6]. Observational studies include case reports and case series; ecologic studies, cross-sectional studies, case-control studies (nested case-control study and case-cohort study); and cohort studies [6]. Experimental studies include clinical trials (randomized, non-randomized, placebo-controlled, open-label, cross-over, factorial); field trials, and community trials [6]. 

**Figure 1:**
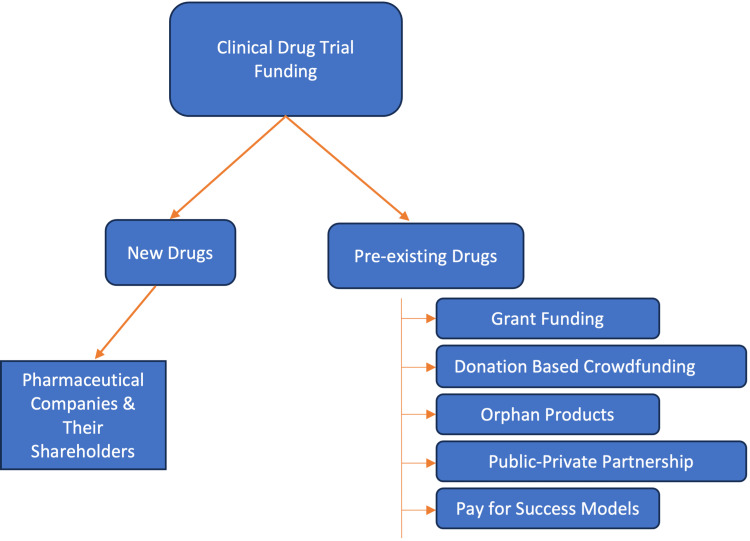
Clinical Drug Trial Funding Flowchart

Once the type of clinical research is determined, the logistics of who runs and is involved in the clinical research is an important consideration. In new drug development, pharmaceutical companies (and their shareholders) are often the sponsors of these studies to bring drugs through the rigorous FDA approval processes [7] (Figure 1). For pre-existing drugs, the funding can incorporate several models including grant funding, donation-based crowdfunding, orphan products, public-private partnerships, and pay-for-success models [7] (Figure 1). 

For the purposes of this review, we will focus on the process of starting clinical drug trials in the context of a private dermatologic practice. There are different phases in clinical drug trials: phase 0 (exploratory study); phase I, phase Ia and phase Ib (non-therapeutic trial); phase II, phase IIa, and phase IIb (exploratory trial); phase III (therapeutic confirmatory trial); and phase IV (post-approval study) [2]. Private practices can be a part of any phase of the drug trial process depending on their resources and prior experiences.

**Figure 2:**
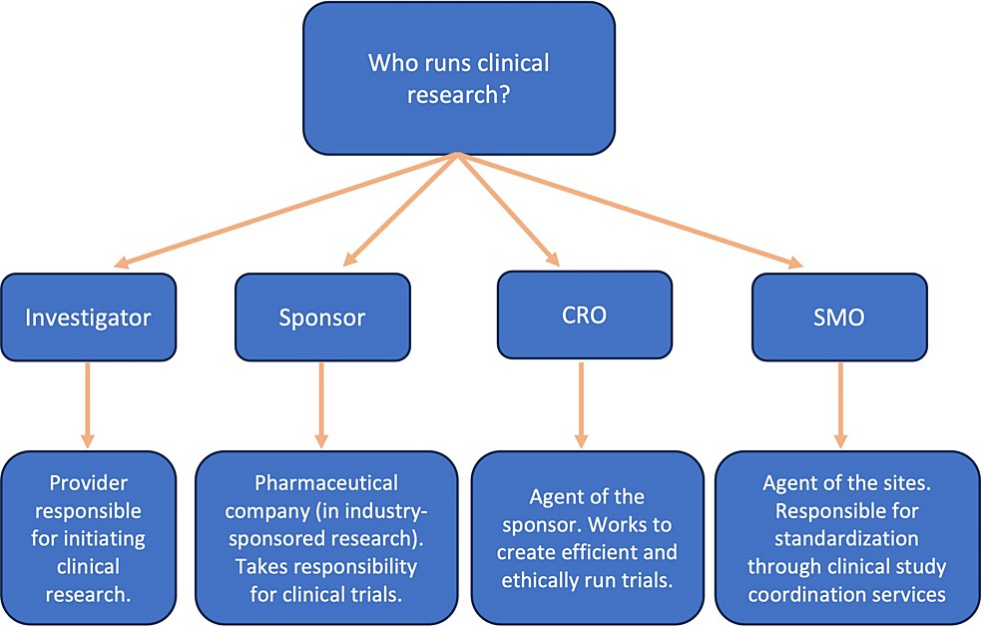
Roles in Clinical Research Flowchart

Understanding roles in clinical research is important. These roles are outlined in Figure 2. In the case of industry-sponsored research, the investigator is the provider responsible for initiating clinical research [2]. The sponsor in the case of industry-sponsored research is the pharmaceutical company or organization providing funds to conduct the research [2]. The sponsor is the entity that takes responsibility for the clinical trials. The contract research organization (CRO) is an agent of the sponsor, working to create efficient and ethically run trials [8]. CROs monitor protocol compliance, clinical data, and ensure that regulations and regulatory documents are managed appropriately [8]. CROs also act on behalf of the sponsor as a liaison between sponsors and clinical sites [8]. Site management organizers (SMOs) conversely are agents of the sites responsible for standardization through clinical study coordination services [9]. Each of these positions is vital to providing the appropriate roles and infrastructure to ensure efficient clinical trials, with the sponsor-related groups being focused on the best quality data to support their product’s development and eventual approval.

Integrating clinical trials into practice requires understanding what will be measured, how we contact and recruit potential trial candidates, protocol reviews, and budgeting. An experienced investigator can develop a long-range approach to the clinical trials by noting what phase the drugs are in, considering the infrastructure of the practice, and how many trials can be conducted at the site without compromising the quality and integrity of data or patient safety. Choosing what assessment tools are best for the study is disease state-specific and may also be dependent on the mode of therapy studied (topical vs oral vs subcutaneous vs intravenous). Assessment tools are often part of the inclusion or exclusion criteria to participate in a clinical trial. These measures include commonly used tools like Eczema Area and Severity Index (EASI) [10] and psoriasis area and severity index (PASI) [11] which are validated, consistent, and can serve as longitudinal as well as cross-sectional data sources to monitor disease burden across patient populations. Another example of a disease-nonspecific dermatologic tool that is often used and applicable to clinical trials is body surface area (BSA), a measure in which each palm-sized amount of the patient correlates to 1% coverage of the body [11]. Other disease processes have other measurements. These can include more specialized burden of disease tools as well as various quality of life and healthcare economic indices (as these are parameters that may capture costs of non-study-related items like emollients, diagnostic studies, and transportation which can all show reductions with successful new interventions). Many studies include a variety of patient-reported outcomes (PROs) to quantify how study conditions may impact the subjects’ sense of well-being.

Protocol development is an essential part of the research process [2]. This process is initiated by the sponsor with FDA feedback and follows established guidelines for randomization, data collection, and statistical powering and must include submission/approval of an institutional review board (IRB) prior to subject recruitment or treatment [2]. The protocol should be detailed, confidential, and feasible [2].

**Figure 3:**
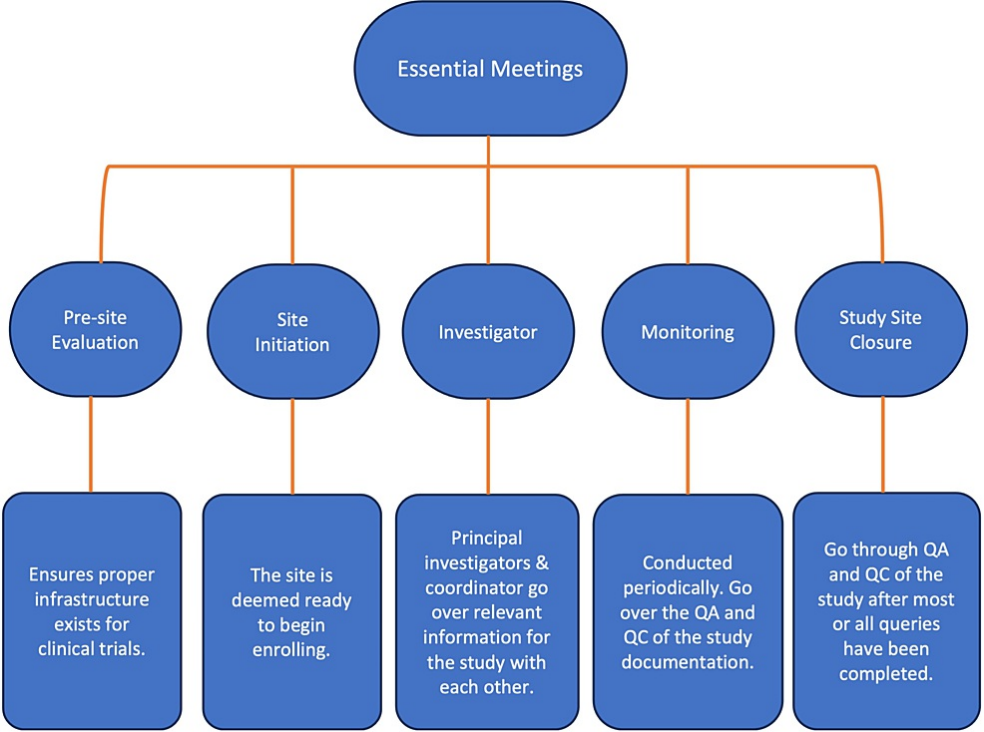
Key Meetings Flowchart

To establish clinical drug trials in private practice several key meetings are integral to the process of initiating a clinical trial (Figure 3). These include the following essential meetings: the pre-site evaluation, site initiation, monitoring, investigator, and finally study site closure meeting. The first meeting is the pre-site evaluation, which is a time in which the practice is evaluated to ensure that the proper infrastructure exists to carry out the specific clinical trial in question. This includes ensuring there are facilities to support the clinical research, review of the protocol, assessment of the availability of potential study subject pool, initiation of contracts, budgeting, assessment tool training (most being through web-based, third-party services), and IRB approval. Small independent practices will have the opportunity to use the sponsor’s preferred central IRB which is much easier and faster to get approvals compared to in-house IRBs required by larger healthcare systems and universities. The second meeting of importance is the site initiation visit. At this visit, all relevant and necessary documents, equipment, and site-sponsor and site-monitor introductions are completed and the site is deemed ready to begin enrolling [1]. The investigator meeting(s) is where many principal investigators and study coordinators will gather for a formal review of all study procedures, site visit details, and any ancillary support services (e.g. radiology, photography, laboratories, etc.) to allow principal instructors and coordinators to go over information relevant to the study and the study protocol with each other [1]. The final type of meetings are the monitoring and study closeout meetings. In these meetings, the site monitor checks the quality assurance (QA) and quality control (QC) of the study documentation periodically and at the end of the study after most or all queries have been completed. 

As mentioned earlier, it is important to understand the mechanics of marketing and recruiting in pharmaceutical trials. Patients can be recruited in several ways including directly from the practice’s patient pool both offline and online, options by referral (offline), or through marketing and targeting strategies (online) [12]. Outside marketing has become an important part of recruitment in dermatologic clinical research with the rise of social media [13]. Outside marketing online through targeted social media advertising has provided ways in which previous obstacles that could be difficult to overcome in research recruitment such as diversity in recruitment, and time and cost reduction in enrollment can be effectively navigated, adding to the accessibility of research recruitment [13]. While social media is a seemingly excellent recruitment method, a caveat to its use for recruitment is the potential for unintended exclusion of older generations who are not active on social media [13]. Through social media, targeting can provide recruitment that more effectively reaches an affected audience that may be able to participate in clinical drug trials [13]. Through methods such as online surveys, algorithms, and AI, recruitment can also benefit by filtering out participants who would not be a good candidate for the clinical trial and allowing candidates that require further screening [13]. 

Another important part of clinical trial research is compensation and its ethical implications. In the US there are existing protections for vulnerable groups (namely children and prisoners) in the context of coercion or undue influence given the socioeconomic and vulnerability status of these groups [14]. In research, the concept of coercion and undue influence interplays with vulnerability status and payment in that a cost too appealing and/or an interplay between low socioeconomic status and/or vulnerability status could affect the patient’s ability to make decisions in a fair, uncompromised way, which threatens the ability to provide informed consent [14]. Although, at the same time it is important to provide fair compensation to subjects involved in clinical trials in part to reimburse for travel, meals, etc., and pay for the time commitment [14]. 

These considerations were legally addressed in 1979 with the creation of the Belmont Report, a document centered around ethics and providing protections to individuals participating in research [15]. In this document, several important concepts are outlined including three major ethical principles: respect for persons, beneficence, and justice, as well as the application of these principles through informed consent, assessment of risk and benefits in research, and the guidelines around the selection of subjects [15]. The first ethical principle, respect for persons, encompasses the concept of autonomy and the protection of subjects with limited autonomy [15]. According to the Belmont Report the second ethical principle, beneficence, is considered to be composed of two main components: “do no harm” and “maximize possible benefits and minimize possible harms” [15]. Lastly, the third ethical principle is justice, which encompasses the concept of equal treatment [15]. These concepts outlined in detail in the Belmont Report are the foundation of our ethical guidelines in research, to safeguard against the inhumane treatment of research participants. 

More recently, the Good Clinical Practice (GCP) quality standard was implemented in 1997 as an international ethical and scientific quality standard [16]. The purpose of this standard is to ensure that results are credible and that the protections of clinical trial participants are adhered to [16]. There are 13 main principles that collectively require that research follow ethical principles, protect participants in clinical trials through informed consent, and maintain confidentiality. It also details that protocols are to be well-defined and thorough, that credible science is to be used, and that there should be more benefits than risks to the participant in a trial [16]. Those conducting research must be qualified and experienced and records should be accessible [16]. Additionally, Good Manufacturing Practice guidelines are to be followed for the production of investigational products [16]. Overall, the GCP standards have brought about an understanding that clinical trials need regulation and have furthered ethical and protective measures for clinical trial subjects and scientific data [16]. 

All these systems are in place to create a structure that allows for clinical trials to run smoothly. On a day-to-day basis, there are practical considerations that allow for smoothly run clinical trials. Some considerations that can make clinical trials easier to manage include standard protocols, source documents, and clinical trial management systems (CTMS). The standardization of protocols not only improves flow but also safeguards against errors and protects the integrity of datasets. Additionally, good documentation practices in source documentation are an essential part of a well-run clinical trial. Source documents are medical records providing documentation prior to, during, and following the clinical trial [17]. These should be detailed, documenting the trial and providing enough information to reconfirm the data if needed [17]. CTMS is the software used to manage and run clinical trials [18]. Having integrative and functional CTMS software is essential as it is a part of each step of the clinical trial process [18]. 

The practical flow of clinical trials requires well-trained staff. In the pre-trial enrollment period, staff often are responsible for pre-screening phone calls or following up with patients from online screening services to make an official in-office screening appointment. At this time they can clarify the pre-screening guidelines to ensure that the patient is eligible for the study. In person, the physician conducts the official screening appointment. If the patient is eligible for the study and the patient provides informed consent by signing the consent form, they move into the screening period, where bloodwork and other potential disqualifiers continue to be monitored. Despite not having a drug administered at this time, if a patient has a screen come back positive after signing the informed consent enrolling them in the trial, this makes them ineligible for the study. This finding must be reported as a potential adverse drug reaction. Once the patient has begun receiving the drug, the appointments largely run through the clinical staff. They are responsible for recording findings, taking photos, and following the protocol during these visits. The visits are spaced according to the protocol schedule. Because of this reliance on staff, it is important that responsible, consistent, and well-trained staff are involved in clinical trials. 

Networking with other physicians can also be a useful tool in establishing clinical trials. Having a physician network that is aware that clinical trials are being conducted and what offices they are conducted at gives an excellent opportunity for external, offline referrals to trials. Creating a physician network, especially in areas that have limited access to care, can bridge a much-needed gap in access to clinical trials. In cases where a patient has failed standard therapies and would otherwise go untreated because of a lack of effective options, clinical trials can offer potential relief for difficult medical conditions. Having a large physician network can also help create diversity in the clinical trial. Patients from different areas are more likely to be reached with a large physician network. 

The landscape of clinical research, its establishment in practice, and its implementation are likely to evolve in the future as our technological capabilities expand. Artificial intelligence (AI) has the capacity to affect recruitment through algorithms and corresponding targeted advertisements and is already used across medical specialties through various algorithms [19]. 

## Conclusions

Incorporating dermatologic research into a private practice can be a daunting task. Although there is a significant need for additional practices providing clinical research opportunities, namely clinical drug trials, it can be overwhelming to begin the process of establishing clinical research. Our aim with this review is to provide an overview of important factors and considerations for participating in clinical research, effectively creating a more accessible environment to incorporate dermatologic clinical research into the private practice setting. However, there is still a need for further research to narrow the gap and create the most efficient routes in incorporating clinical research effectively. The areas we outlined are some of the most important to focus on when establishing clinical drug trials, and will become a large part of day-to-day life in private practices offering clinical trials. With this review we hope to encourage and provide guidance to other private practices who are looking to enter into clinical trials.
